# Time-Varying Risk Factors for Incident Fractures in Kidney Transplant Recipients: A Nationwide Cohort Study in South Korea

**DOI:** 10.3390/jcm12062337

**Published:** 2023-03-17

**Authors:** Sang Hun Eum, Da Won Kim, Jeong-Hoon Lee, Jin Seok Jeon, Heungman Jun, Jaeseok Yang, Myoung Soo Kim, Hye Eun Yoon, on Behalf of the Korean Organ Transplantation Registry Study Group

**Affiliations:** 1 Division of Nephrology, Department of Internal Medicine, Incheon St. Mary’s Hospital, College of Medicine, The Catholic University of Korea, Incheon 21431, Republic of Korea; trickyspot@gmail.com (S.H.E.); funkywizz@naver.com (D.W.K.); 2 Department of Surgery, Myongji Hospital, Hanyang University, Goyang 10475, Republic of Korea; jhunlee69@gmail.com; 3 Department of Internal Medicine, Soonchunhyang University Seoul Hospital, Seoul 04401, Republic of Korea; jeonmark@naver.com; 4 Department of Surgery, Korea University Anam Hospital, Seoul 02841, Republic of Korea; midasia@hanmail.net; 5 Department of Internal Medicine, Yonsei University College of Medicine, Seoul 03722, Republic of Korea; jcyjs@yuhs.ac; 6 Department of Surgery, Yonsei University College of Medicine, Seoul 03722, Republic of Korea; ysms91@yuhs.ac

**Keywords:** CKD-MBD, fracture, kidney transplantation, bone disease, immunosuppression

## Abstract

Little is known about the time-varying risk factors for fractures in kidney transplant recipients (KTRs). Using the Korea Organ Transplantation Registry, a nationwide cohort study of KTRs, the incidence, locations, and time-varying predictors of fractures were analyzed, including at baseline and post-transplant 6-month variables in KTRs who underwent KT between January 2014 and June 2019. Among 4134 KTRs, with a median follow-up of 2.94 years (12,441.04 person-years), 63 patients developed fractures. The cumulative 5-year incidence was 2.10%. The most frequent locations were leg (25.40%) and foot/ankle (22.22%). In multivariable analysis, older recipient age at baseline (hazard ratio [HR], 1.035; 95% confidence interval [CI], 1.007–1.064; *p* = 0.013) and higher tacrolimus trough level (HR, 1.112; 95% CI, 1.029–1.202; *p* = 0.029) were associated with higher risks for fractures. Pretransplant diabetes mellitus had a time-dependent impact on fractures, with increasing risk as time elapses (HR for diabetes mellitus 1.115; 95% CI, 0.439–2.832; HR for diabetes mellitus × time, 1.049; 95% CI, 1.007–1.094; *p* = 0.022). In conclusion, KTRs had a high risk of peripheral skeletal fractures in the first 5 years. At baseline recipient age, pretransplant diabetes mellitus and tacrolimus trough level after KT were responsible for the fractures in KTRs.

## 1. Introduction

Previous studies have described that the incidence of fractures increases in kidney transplant recipients (KTRs) versus the general population [1] and the risk exceeds that in dialysis patients in the first 1 to 3 years after transplantation [2]. These findings suggest that the bone remains fragile despite improvements in bone and mineral disturbances following kidney transplantation (KT). Chronic kidney disease (CKD) patients have chronic kidney disease–mineral bone disease (CKD-MBD), a disorder of mineral and bone metabolism. CKD-MBD encompasses biochemical abnormalities of calcium, phosphorus, parathyroid hormone (PTH), vitamin D, bone disease, and vascular calcification, which all contribute to fractures [3]. Preexisting CKD-MBD, consequences of KT-specific therapies including glucocorticoid use, and progressive graft dysfunction after KT contribute to high risks of fractures [4].

Several large-scale studies have examined factors that predispose KTRs to fractures [5,6]. However, the incidence of fractures varies widely from 3.3 to 99.6 fractures per 1000 person-years, with a 5-year cumulative incidence of 0.85–27% [5]. This is because the patient characteristics, study quality, the definition of fractures, and follow-up duration differed among studies. In addition, the changing patterns of treatments, such as the corticosteroid-free regimen adopted after 2000 and the increase in mean recipient age, were not reflected in recent studies. Thus, existing studies have poor consensus on general risk factors and transplant-specific risk factors for fractures in KTRs. Most of the previous studies included baseline variables when investigating predictors of fractures. However, the effect of a fixed baseline variable may change over time, or the risk factor itself may change over time [7]. After KT, laboratory variables change as the allograft function recovers and the early post-transplantation period accompanies the greatest change in immunosuppressants. The present study aimed to investigate the incidence of and assess the time-varying risk factors for fractures following KT by including baseline and post-transplant variables in the analysis.

## 2. Materials and Methods

### 2.1. Study Population and Data Collection

Data were obtained from the Korea Organ Transplantation Registry (KOTRY), a prospective multicenter nationwide cohort study of KTRs in South Korea. Forty-one transplantation centers participated in the KOTRY. In the KOTRY, pretransplant and follow-up data are collected by each center at baseline and post-transplantation after 6 months and 1 year, and then annually. Therefore, the collected dataset is complete for each participant. A total of 5403 KTRs aged ≥ 18 years who underwent KT between January 2014 and June 2019 were enrolled in the KOTRY. Among them, 1269 KTRs lacked post-transplantation 6-month data because their post-transplant periods were less than 6 months. Therefore, this study included 4134 KTRs in the analysis. The KOTRY provided patient demographics at the time of transplantation, including age, sex, height, weight, body mass index (BMI), smoking status, primary renal disease, date of dialysis initiation, dialysis modalities used before KT, dialysis vintage, KT date, donor type, other medical comorbidities, and individual patient treatments following KT. BMI was calculated as the patient’s weight in kilograms divided by height in meters squared (kg/m^2^). Baseline laboratory parameters included serum levels of hemoglobin, creatinine, albumin, corrected calcium (Ca), phosphorus (P), and intact PTH at the time of transplantation. Serum levels of hemoglobin, creatinine, albumin, corrected Ca, and P, and tacrolimus trough levels at 6 months after KT were collected. Data on medications taken at 6 months post-transplantation including vitamin D analogs, tacrolimus, cyclosporine, mycophenolic acid, mammalian target of rapamycin (mTOR) inhibitor, and corticosteroids were also collected. The estimated glomerular filtration rate (eGFR) was calculated using the Chronic Kidney Disease Epidemiology Collaboration equation [8]. Death-censored graft loss and rejection treatment within 6 months post-transplantation were also included.

The outcome of this study was fracture occurrence. The information on fracture occurrence was collected in the original registry, which was identified by medical records from patients’ charts. All incident fractures were identified regardless of cause, and traumatic fractures were not assessed separately according to trauma level, as previously described [9]. This is because judging the level of trauma is subjective, as fractures at all locations were included, and high trauma non-spine fractures were also associated with a low bone mineral density as low trauma non-spine fractures [10].

All patients provided written informed consent before KOTRY enrollment. The study was performed in line with the principles of the Declaration of Helsinki and approved by the Institutional Review Board of Incheon St. Mary′s Hospital (OC19OISI0172).

### 2.2. Comparison of Clinical Characteristics and Cumulative Incidence of Fractures

Patients were divided into two groups: those who developed fractures (fracture group) and those who did not develop fractures (no fracture group). We compared the clinical baseline characteristics between the two groups, including demographic characteristics, laboratory findings, use of vitamin D analogs, and transplant-specific factors such as percentage of panel reactive antibody, the presence of a human leukocyte antigen donor-specific antibody, the positivity of cross-match testing, type of induction therapy (interleukin [IL]-2 receptor antibody or anti-thymocyte globulin), and main immunosuppressant type (tacrolimus, cyclosporine, mycophenolic acid, mTOR inhibitor, and corticosteroid). Laboratory parameters and medication use at 6 months post-transplantation and death-censored graft loss and rejection treatment within 6 months of post-transplantation were compared between the two groups. The cumulative incidence of fractures was also compared between the two groups.

### 2.3. Statistical Analysis

Continuous data are expressed as mean ± standard deviation or median with interquartile range, and comparisons were made using Student′s *t*-test or the Mann–Whitney U-test as appropriate. Categorical data are expressed as numbers with percentages and were compared using the chi-square test. The time to the first fracture was modeled using the Kaplan–Meier method. The Cox proportional hazard (PH) model was used to identify risk factors for fractures. The univariable analysis was followed by multivariable analyses using the forward conditional method. To determine the variables to be included in the multivariable model, the univariable Cox PH regression analysis is applied first to identify the impact of individual variables. Variables are identified as significant using a 0.2 significance level in the univariable analysis. To test the assumption of proportionality after the construction of the multivariable model, the scaled Schoenfeld residuals have been used. If the model displayed non-proportionality for variables included, the stratified Cox PH model and time-dependent Cox PH model were used to identify baseline and 6-month post-transplantation risk factors for fractures [11]. The results are presented as hazard ratios (HR) with 95% confidence intervals (CI). In all analyses, a *p*-value < 0.05 (two-tailed) was considered to indicate statistical significance. The statistical analyses were performed using R statistical software (version 4.1.3; R Foundation for Statistical Computing, Vienna, Austria). In addition, all graphs were generated using Prism software (GraphPad, San Diego, CA, USA) and R statistical software.

## 3. Results

### 3.1. Patient Characteristics at Baseline

Table 1 shows the baseline characteristics of the patients by study group. The mean recipient age was 49.00 years and the mean BMI was 23.09 kg/m^2^. Among the 4134 KTRs, 59.19% were male, 22.62% were smokers, and 28.96% had diabetes mellitus. The median time on dialysis was 30.33 months [interquartile range (IQR), 3.87–85.25 months]; 14.85% of the patients underwent preemptive transplantation, while 7.89% of the patients underwent re-transplantation.

The fracture group was older and had a higher prevalence of diabetes mellitus and diabetic nephropathy as the primary renal diseases than the no fracture group. The fracture group had lower baseline intact PTH levels, more frequently used IL-2 receptor antibody, and less frequently used anti-thymocyte globulin as an induction regimen than the no fracture group.

### 3.2. Patient Characteristics at 6 Months Post-Transplantation

Laboratory data at 6 months post-transplantation were compared between the two groups. The fracture group had a lower serum P level and Ca × P product value. Tacrolimus was used more frequently as the maintenance immunosuppressant than the no fracture group, although it was not statistically significant. There were no significant differences in serum levels of hemoglobin, albumin, and corrected Ca, eGFR, proportions of death-censored graft loss, and rejection treatment within 6 months, and medications including vitamin D analogs, cyclosporine, mycophenolic acid, mTOR inhibitor, and corticosteroids. Despite the tendency of a lower tacrolimus dose in the fracture group, the serum tacrolimus trough level was significantly higher in the fracture group (Table 2).

### 3.3. Fracture Incidence and Location

**Figure 1:**
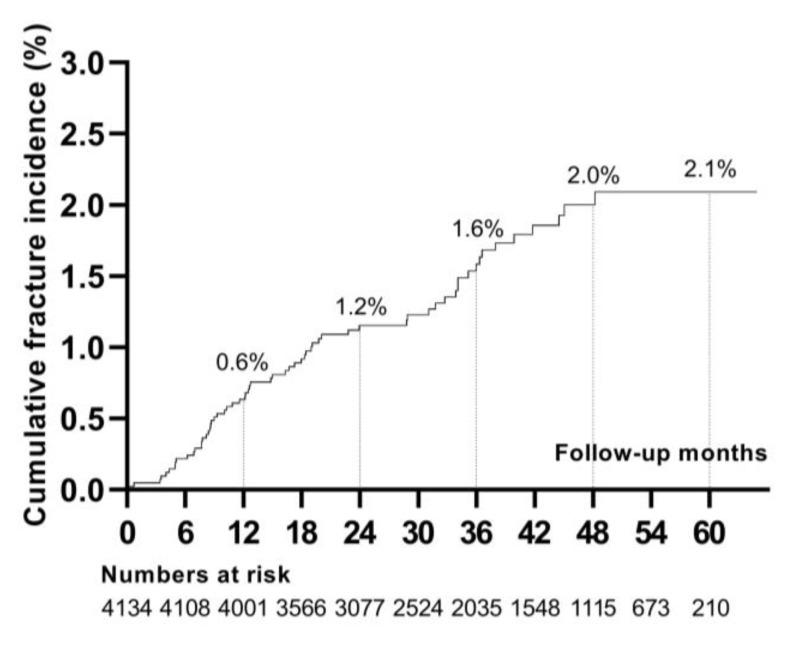
Cumulative incidence of fractures after kidney transplantation.

**Figure 2:**
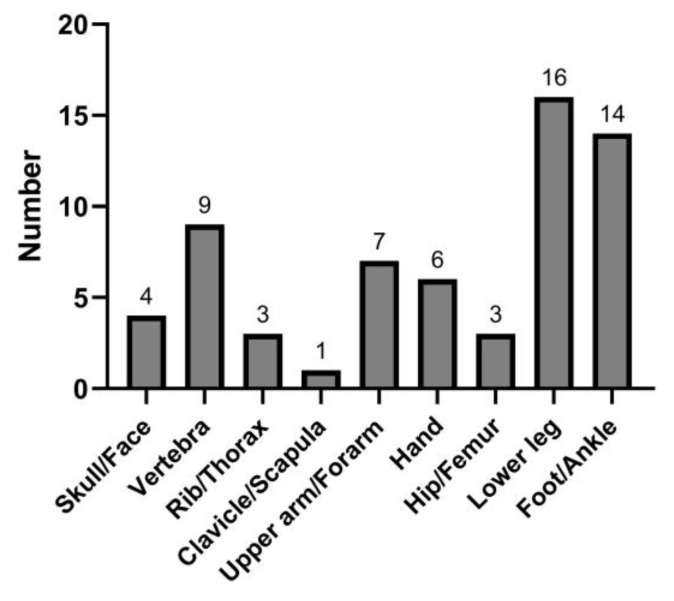
Location of the incident fractures.

During a follow-up of 12,441.04 person-years (median, 2.94 years), 63 patients developed incident fractures. The cumulative incidence of fractures was 2.10% at 5 years (Figure 1). The most frequent fracture locations were the lower leg [n = 16 (25.40%)] and foot/ankle [n = 14 (22.22%)]. Less common fracture sites were the vertebra [n = 9 (14.29%)], upper arm/forearm [n = 7 (11.11%)], hand [n = 6 (9.52%)], skull/face [n = 4 (6.35%)], rib/thorax [n = 3 (4.76%)], hip/femur [n = 3 (4.76%)], and clavicle/scapula [n = 1 (1.59%)] (Figure 2).

### 3.4. Predictors of Incident Fractures

**Figure 3:**
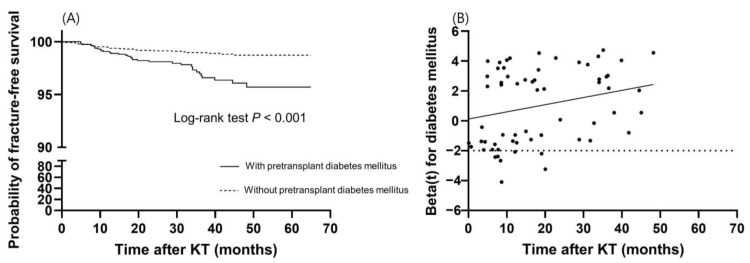
Time-dependent effect of pretransplant diabetes mellitus on fractures. (**A**) Kaplan–Meier survival curve of fracture-free survival according to pretransplant diabetes mellitus. (**B**) The effect of pretransplant diabetes mellitus on fractures over time. The dotted line is the reference line for the null effect. The solid line shows a linear effect with a slope of 0.048.

Risk factors for incident fractures were analyzed (Table 3 and Table 4). In the univariable analysis, fracture risk was influenced by recipient age, pretransplant diabetes mellitus, preemptive KT, and the use of IL-2 receptor antibody and anti-thymocyte globulin as induction therapy. Among the variables at 6 months post-transplantation, serum P level, Ca × P product, and tacrolimus trough level were associated with incident fractures in the univariable analysis. In multivariable analysis, since pretransplant diabetes mellitus violated the proportional hazards assumption with a *p*-value of less than 0.05 by scaled Schoenfeld residuals, we applied the stratified Cox regression (Table 3). Recipient age (HR, 1.035; 95% CI, 1.007–1.064; *p =* 0.013) and tacrolimus trough level (HR, 1.112; 95% CI, 1.028–1.202; *p* = 0.008) were significantly associated with a higher risk of fractures. Furthermore, to identify the time-varying effect of pretransplant diabetes mellitus, we performed an extended Cox regression analysis with diabetes mellitus as a continuous time-varying coefficient (Table 4). In this multivariable model, recipient age (HR, 1.035; 95% CI, 1.007–1.064; *p* = 0.013) and tacrolimus trough level (HR, 1.112; 95% CI, 1.029–1.202; *p* = 0.008) were significantly associated with a higher risk of fractures. The fracture-free survival rate of KTRs with pretransplant diabetes mellitus was significantly lower than that of KTRs without pretransplant diabetes mellitus (*p* < 0.001), and the survival curve declined steeper as the time after KT elapsed (Figure 3A). Pretransplant diabetes mellitus showed an increased hazard ratio over the length of the follow-up period (HR, 1.049; 95% CI, 1.007–1.094; *p* = 0.022) (Table 4 and Figure 3B).

## 4. Discussion

This study demonstrated the time-varying tacrolimus trough level and time-varying effect of baseline diabetes mellitus affected the risk of fractures in KTRs. In this nationwide cohort study of 4134 KTRs, the 5-year cumulative incidence of fractures after KT was 2.10%. The most common fracture sites were the leg and foot/ankle. Older recipient age at baseline and higher tacrolimus trough level at 6 months post-transplantation increased the risk of fractures, and pretransplant diabetes mellitus showed an increased risk of fractures as time elapsed.

Previous reports demonstrated that KTRs are more susceptible to fractures in the early rather than late post-transplantation period. KTRs had a 1.34-fold greater risk of hip fractures than dialysis patients in the first 3 years after transplantation, and the risk decreased to a level comparable to that of dialysis patients after 3 years [2]. Similarly, the fracture risk of KTRs was up to 4.6-fold higher than that of the general population in the first 3 years after KT [12]. These findings suggest that KT does not restore the bone fragility of preexisting CKD-MBD, despite the improvement in mineral metabolism disturbances [13]. As vertebral fractures were closely related to mortality in CKD patients [14] and KTRs with fractures were more likely to experience graft loss or mortality [15], fractures continue to be a significant problem after KT. The cumulative incidence of fractures ranged widely from 0.85% to 27% in previous studies [5]. A recent study reported a 3-year cumulative incidence of non-vertebral fractures of 1.6% and a 10-year cumulative incidence of 1.7% [16], consistent with the results of this study. Although fracture incidence of KTRs was reported in various studies, the relatively low incidence in this study can be explained by the emergence of corticosteroid-limiting or withdrawal protocols after 2000 [17] and advances in the management of CKD-MBD that persist after transplantation.

In the present study, the foot/ankle and lower leg were the most common fracture locations, a finding consistent with those of previous reports [9,18]. The high incidence of fractures in the peripheral skeleton, an atypical site of osteoporotic fractures, suggests that various factors are involved in post-transplantation bone fragility. Pre-transplant renal-specific bone factors, post-transplant changes caused by immunosuppressive therapy, gradual allograft dysfunction, and continued mineral metabolism disturbances may impact peripheral fractures [19]. Vitamin K deficiency is also known to affect vascular calcification and poor bone quality in CKD patients, and it was also associated with incident fractures in de novo KTRs [20,21]. Immunosuppressive treatments alter the bone structure, function, and formation in combination with persistent secondary or tertiary hyperparathyroidism [22]. Glucocorticoid-induced osteopenia occurs because of impaired osteoblastogenesis and early osteoblast apoptosis, affecting the trabecular bone of the axial skeleton in particular [23]. In addition, the cumulative dose of glucocorticoids negatively correlates with bone turnover and volume [22]. In murine models, cyclosporine stimulated osteoclast activity more than osteoblast activity, resulting in bone loss [22,24], while tacrolimus induced severe trabecular bone loss [25]. An in vitro study showed that sirolimus interfered with osteoblast proliferation and differentiation [26], while everolimus reduced cancellous bone loss in ovariectomized rats by decreasing osteoclast-mediated bone resorption [27]. The susceptibility of the peripheral skeleton to fractures was demonstrated in a study using an early corticosteroid withdrawal protocol [28]. Interestingly, the bone mineral density in the peripheral skeleton showed progressive deterioration despite early corticosteroid withdrawal, whereas cortical bone mass and strength in the central skeleton were preserved. This is due to persistent hyperparathyroidism and elevated remodeling rates, which result in cortical and trabecular losses and decreased bone strength in the peripheral skeleton [28].

In this study, older age and diabetes mellitus at baseline were risk factors for fractures, as reported by previous studies [5,6,12,29]. However, dialysis modality, dialysis vintage, deceased donor, and female sex were not associated with fractures, a finding that is inconsistent with those of other reports [5,12,30]. In South Korea, most end-stage kidney disease patients are receiving hemodialysis and the proportion of peritoneal dialysis accounts for less than 10% in the past 10 years [31]. The effect of dialysis modality on fractures may not be clearly determined because of the low number of peritoneal dialysis patients. Recent studies also reported that donor type is not significantly associated with fracture risk [6,15]. As this study lacks data on menopausal status, estrogen therapy, or specific osteoporosis treatments, the reason for the lack of an association between the female sex and fractures is unclear. Since this study included KTRs performed in 2014–2019, these patients might have been treated with different drugs for CKD-MBD and osteoporosis than those in the early 2000s. Advances in drugs for CKD-MBD and osteoporosis might have weakened the effect of the female sex on fractures. The reason for the insignificant effects of dialysis vintage on fractures in this study is also unclear. We speculate that the different populations and study periods may have affected these discrepant results.

Interestingly, pretransplant diabetes mellitus showed an increased hazard ratio over the follow-up period. Diabetes mellitus is a well-known risk factor for fractures, even in the absence of kidney disease. Proposed pathophysiologic mechanisms are the direct effects of chronic hyperglycemia on bone microarchitecture, inefficient distribution of bone mass, and insufficient repair and adaptation response of bone. Increased risk of falls because of visual impairment and neuropathy may all increase the risk of fractures in patients with diabetes mellitus [32]. In a recent study using the US Renal Data System, the risks for upper extremity and lower leg fractures were significantly higher in dialysis patients with diabetic nephropathy than those with other renal diseases [33]. Since hyperglycemia worsens with the use of calcineurin inhibitors and corticosteroids, the ongoing effect of hyperglycemia on bone health may increase the risk for peripheral skeleton fractures, as post-transplantation periods increase in KTRs with diabetes mellitus.

In this study, we aimed to evaluate the effect of time-varying factors on fractures since various factors change after KT, including allograft function, mineral metabolism, and immunosuppressants. Phosphate is a major factor in CKD-MBD, and hyperphosphatemia is closely related to fractures in CKD patients [34]. Conversely, in KTRs, hypophosphatemia was associated with increased fracture risk [35]. In this study, KTRs with fractures had a significantly lower serum phosphorus level at 6 months post-transplantation compared to KTRs without fractures. However, hypophosphatemia at 6 months post-transplantation did not predict future fractures in this study. Among the post-transplantation variables, a higher tacrolimus trough level at 6 months post-transplantation was associated with an increased risk of fractures. As tacrolimus is bound to plasma proteins in the circulation, especially albumin, circumstances affecting plasma protein concentration may change the tacrolimus level [36]. Since our data did not include long-term tacrolimus levels, the results are insufficient to conclude that long-term high tacrolimus levels directly impact bone health. However, there is evidence that tacrolimus affects bone structure in the current literature. In rat models, calcineurin inhibitors, cyclosporine, or tacrolimus, stimulate bone mass loss independent of corticosteroid treatment [37]. It was shown that tacrolimus induced trabecular bone loss and high-turnover osteoporosis in rats [25]. In comparison with cyclosporine, the reduction in trabecular bone mass was more marked in tacrolimus-treated rats [38]. In a study with corticosteroid withdrawal protocol, there was a progressive decline in cortical and trabecular bone density at the peripheral skeleton, despite of preservation of bone mineral density at the central skeleton [28]. Thus, higher tacrolimus trough level may have contributed to a more severe trabecular bone loss at the peripheral skeleton in KTRs.

This study has several limitations. First, confounders associated with fractures such as previous fractures, alcohol consumption, physical fitness, exercise, menopausal status, estrogen replacement therapy, intact PTH level at 6 months post-transplantation, treatment for hyperparathyroidism, including parathyroidectomy, and cumulative doses of corticosteroids were not analyzed, nor was dual X-ray absorptiometry used. Especially, the lack of data on cumulative doses of corticosteroids might have underreported the detrimental effects of steroids on bone fractures in this study. Second, the fracture events may have been underestimated; the incidence of vertebral fractures may have been missed because non-traumatic vertebral fractures can be undiagnosed unless actively searched for. Third, the long-term time-varying variables beyond 1 year after KT were not analyzed because of insufficient data. Fourth, defining fractures according to the level of trauma could not be conducted. However, the current study has strengths, including being a nationwide cohort study that included relatively recent KTRs and using complete data from comprehensive medical records. In addition, medications and laboratory parameters at 6 months post-transplantation were included to assess the time-varying effect of certain factors and the effects of time-varying variables, which were not evaluated in previous studies.

In conclusion, KTRs are susceptible to peripheral skeletal fractures in the first 5 years. Baseline recipient age and pretransplant diabetes mellitus were associated with fractures in KTRs, and the strength of association with diabetes mellitus increased over the follow-up period. A tacrolimus trough level after KT may be associated with the risk of fractures, which needs verification with tacrolimus levels in the long term. A better understanding of the incidence of and risk factors for fractures remains important, as a well-established fracture prediction tool can prevent and guide treatment decisions in KTRs.

## Figures and Tables

**Table 1:** Baseline characteristics of the kidney transplant recipients.

	Total (*n* = 4134)	No Fracture (*n* = 4071)	Fracture (*n* = 63)	*p*
Recipient age (years)	49.00 ± 11.50	48.92 ± 11.51	54.18 ± 9.49	<0.001
Recipient sex, male (%)	2447 (59.19)	2405 (59.08)	42 (66.67)	0.224
Donor age (years)	46.64 ± 13.03	46.65 ± 13.03	45.87 ± 13.05	0.640
Donor sex, male (%)	2201 (53.24)	2168 (53.25)	33 (52.38)	0.890
Deceased donor (%)	1533 (37.08)	1505 (36.97)	28 (44.44)	0.223
BMI, kg/m^2^	23.09 ± 3.57	23.09 ± 3.57	22.92 ± 3.81	0.700
Smoking (%)	935 (22.62)	920 (22.60)	15 (23.81)	0.804
Pretransplant diabetes mellitus (%)	1197 (28.96)	1164 (28.59)	33 (52.38)	<0.001
Hypertension (%)	3709 (89.72)	3654 89.76	55 (87.30)	0.516
History of CVD (%)	429 (10.38)	419 (10.29)	10 (15.87)	0.150
Primary renal disease (%)				0.003
Diabetes	950 (22.98)	923 (22.67)	27 (42.86)	
Hypertension	654 (15.82)	643 (15.79)	11 (17.46)	
Glomerulonephritis	1373 (33.21)	1361 (33.43)	12 (19.05)	
Others	324 (7.84)	320 (7.86)	4 (6.35)	
Unknown	833 (20.15)	824 (20.24)	9 (14.29)	
RRT before KT				0.052
Hemodialysis (%)	2943 (71.20)	2891 (71.01)	52 (82.54)	
Peritoneal dialysis (%)	523 (12.65)	517 (12.70)	6 (9.52)	
KT (%)	54 (1.31)	52 (1.28)	2 (3.17)	
Preemptive KT (%)	614 (14.85)	611 (15.01)	3 (4.76)	
Dialysis vintage (months)	30.33 (3.87–85.25)	30.33 (3.84–84.91)	31.56 (5.80–61.73)	0.344
Retransplant (%)	326 (7.89)	318 (78.11)	8 (12.70)	0.153
Mean arterial pressure (mmHg)	102.16 ± 14.10	102.19 ± 14.10	100.23 ± 13.74	0.270
Hemoglobin (g/dL)	10.73 ± 1.60	10.73 ± 1.60	10.78 ± 1.48	0.800
Albumin (g/dL)	3.93 ± 0.52	3.93 ± 0.52	3.92 ± 0.56	0.840
Corrected calcium (mg/dL)	9.06 ± 0.89	9.05 ± 0.90	9.11 ± 0.73	0.620
Phosphorus (mg/dL)	5.11 ± 1.54	5.11 ± 1.54	4.99 ± 1.56	0.540
Ca × P product (g^2^/dL^2^)	45.96 ± 14.29	45.97 ± 14.28	45.43 ± 14.80	0.770
Intact PTH (pg/mL)	245.0 (128.0–407.0)	246.0 (128.0–408.0)	138.0 (83.0–316.0)	0.008
Vitamin D analogs (%)	730 (17.66)	722 (17.74)	8 (12.70)	0.279
Panel reactive antibody ≥ 50% (%)	167 (4.04)	166 (4.08)	1 (1.59)	0.319
HLA-DSA (%)	265 (6.41)	261 (6.50)	4 (6.35)	0.984
Positive cross-match (%)	218 (5.27)	215 (5.35)	3 (4.76)	0.855
ABO incompatibility (%)	643 (15.55)	637 (15.65)	6 (9.52)	0.183
Desensitization (%)	922 (22.30)	911 (22.68)	11 (17.46)	0.352
IL-2 receptor antibody (%)	3326 (80.45)	3268 (80.28)	58 (92.06)	0.004
ATG (%)	844 (20.42)	841 (20.66)	3 (4.76)	0.002
Tacrolimus (%)	3989 (96.49)	3926 (96.44)	63 (100.00)	0.172
Cyclosporine (%)	134 (3.24)	134 (3.29)	0 (0.00)	0.270
Mycophenolic acid (%)	3812 (92.21)	3754 (92.21)	58 (92.06)	0.816
mTOR inhibitor (%)	48 (1.16)	47 (1.15)	1 (1.59)	0.524
Corticosteroid (%)	4067 (98.38)	4006 (98.40)	61 (96.83)	0.272
Follow-up months	36.51 (24.23–49.73)	35.77 (24.07–49.56)	46.13 (35.40–55.54)	<0.001

ATG, anti-thymocyte globulin; BMI, body mass index; CVD, cardiovascular disease; RRT, renal replacement therapy; KT, kidney transplantation; Ca, calcium; P, phosphorus; PTH, parathyroid hormone; HLA-DSA; human leukocyte antigen donor-specific antibody; IL-2, interleukin-2; mTOR, mammalian target of rapamycin.

**Table 2:** Clinical characteristics of the kidney transplant recipients at 6 months post-transplantation.

	Total (*n* = 4134)	No Fracture (*n* = 4071)	Fracture (*n* = 63)	*p*
Hemoglobin (g/dL)	12.92 ± 1.80	12.93 ± 1.80	12.61 ± 1.62	0.168
Albumin (g/dL)	4.27 ± 0.37	4.27 ± 0.37	4.23 ± 0.34	0.376
Corrected calcium (mg/dL)	9.39 ± 0.74	9.39 ± 0.74	9.41 ± 0.55	0.840
Phosphorus (mg/dL)	3.23 ± 0.71	3.23 ± 0.71	3.03 ± 0.66	0.024
Ca × P product (g^2^/dL^2^)	30.12 ± 6.07	30.15 ± 6.07	28.40 ± 5.85	0.023
eGFR (mL/min/1.73 m^2^)	67.36 ± 20.02	67.34 ± 20.03	68.99 ± 19.13	0.516
Vitamin D analogs (%)	657 (15.95)	644 (15.88)	13 (20.63)	0.307
Tacrolimus (%)	3919 (94.80)	3856(94.72)	63 (100.00)	0.077
Tacrolimus dose (mg)	4.20 ± 2.41	4.21 ± 2.42	3.64 ± 1.90	0.064
Tacrolimus dose per body weight (mg/10 kg)	0.69 ± 0.42	0.69 ± 0.42	0.60 ± 0.32	0.106
Tacrolimus level (mg/dL)	6.69 ± 2.52	6.68 ± 2.49	7.56 ± 3.65	0.006
Cyclosporine (%)	176 (4.26)	176 (4.32)	0 (0.00)	0.113
Mycophenolic acid (%)	3446 (83.36)	3389 (83.25)	57 (90.48)	0.126
mTOR inhibitor (%)	215 (5.20)	211 (5.18)	4 (6.35)	0.568
Corticosteroid (%)	4036 (97.63)	3974 (97.62)	62 (98.42)	1.000
Corticosteroid dose (mg)	6.99 ± 3.55	6.99 ± 3.53	6.65 ± 4.75	0.455
Death-censored graft loss (%)	14 (0.34)	14 (0.34)	0 (0.00)	1.000
Rejection treatment (%)	698 (16.88)	686 (16.85)	12 (19.05)	0.644

Ca, calcium; P, phosphorus; eGFR, estimated glomerular filtration rate; mTOR, mammalian target of rapamycin.

**Table 3:** Risk factors for incident fractures of the kidney transplant recipients.

	Univariable		Stratified Multivariable	
	HR (95% CI)	*p*	HR (95% CI)	*p*
Variables at baseline				
Recipient age	1.050 (1.024–1.076)	<0.001	1.035 (1.007–1.064)	0.013
Recipient sex, female	0.716 (0.424–1.210)	0.212		
Donor age	0.998 (0.979–1.017)	0.806		
Donor sex, female	1.064 (0.649–1.745)	0.806		
Deceased donor	1.306 (0.794–2.147)	0.293		
BMI (kg/m^2^)	1.010 (0.923–1.062)	0.773		
Smoking	1.065 (0.595–1.904)	0.832		
Pretransplant diabetes mellitus	2.847 (1.736–4.669)	<0.001	–	–
Hypertension	0.779 (0.371–1.634)	0.508		
History of CVD	1.676 (0.853–3.294)	0.134	0.903 (0.444–1.834)	0.777
RRT before KT				
Hemodialysis	1.000 (Reference)		1.000 (Reference)	
Peritoneal dialysis	0.607 (0.261–1.414)	0.248		
KT	2.011 (0.490–8.259)	0.332		
Preemptive KT	0.274 (0.086–0.878)	0.030	0.360 (0.111–1.166)	0.088
Dialysis vintage (month)	1.002 (0.998–1.006)	0.340		
Retransplant	1.646 (0.784–3.455)	0.188	2.102 (0.981–4.506)	0.056
Mean arterial pressure	0.990 (0.973–1.008)	0.282		
Hemoglobin (g/dL)	1.019 (0.873–1.190)	0.811		
Albumin (g/dL)	0.967 (0.603–1.550)	0.888		
Corrected calcium (mg/dL)	1.062 (0.808–1.350)	0.666		
Phosphorus (mg/dL)	0.950 (0.807–1.120)	0.543		
Ca × P product (mg^2^/dL^2^)	0.997 (0.980–1.015)	0.755		
Intact PTH	1.000 (0.999–1.001)	0.478		
Vitamin D analogs	0.676 (0.322–1.420)	0.301		
Desensitization	0.760 (0.397–1.457)	0.409		
IL-2 receptor antibody	4.513 (1.415–14.390)	0.011	1.140 (0.155–8.357)	0.898
Anti-thymocyte globulin	0.203 (0.064–0.646)	0.007	0.160 (0.021–1.200)	0.075
Tacrolimus	N/A	N/A		
Cyclosporine	N/A	N/A		
Mycophenolic acid	1.132 (0.454–2.828)	0.790		
mTOR inhibitor	1.077 (0.149–7.776)	0.942		
Corticosteroid	0.539 (0.132–2.206)	0.390		
Variables at 6 months post-transplantation				
Hemoglobin	0.902 (0.784–1.037)	0.146	0.880 (0.762–1.018)	0.085
eGFR	1.004 (0.991–1.016)	0.574		
Albumin	0.684 (0.348–1.344)	0.271		
Corrected calcium	1.035 (0.743–1.441)	0.839		
Phosphorus	0.651 (0.452–0.938)	0.021	0.717 (0.215–2.398)	0.590
Ca × P product	0.950 (0.910–0.992)	0.020	0.970 (0.844–1.115)	0.667
Vitamin D analogs	1.521 (0.825–2.801)	0.179	1.304 (0.698–2.437)	0.405
Tacrolimus	N/A	N/A		
Tacrolimus dose	0.889 (0.784–1.008)	0.067	0.779 (0.548–1.107)	0.164
Tacrolimus dose per body weight	0.551 (0.271–1.121)	0.100	2.621 (0.368–18.697)	0.336
Tacrolimus trough level	1.121 (1.04–1.208)	0.003	1.112 (1.028–1.202)	0.008
Cyclosporine	N/A	N/A		
Mycophenolic acid	1.853 (0.799–4.296)	0.151	2.222 (0.884–5.588)	0.090
mTOR inhibitor	1.243 (0.451–3.421)	0.674		
Corticosteroid	1.496 (0.207–10.790)	0.689		
Corticosteroid dose	0.970 (0.892–1.054)	0.469		
Death-censored graft loss	N/A	N/A		
Rejection treatment	1.172 (0.625–2.198)	0.621		

BMI, body mass index; CVD, cardiovascular disease; RRT, renal replacement therapy; KT, kidney transplantation; Ca, calcium; P, phosphorus; PTH, parathyroid hormone; HLA-DSA; human leukocyte antigen donor-specific antibody; IL-2, interleukin-2; mTOR, mammalian target of rapamycin; eGFR, estimated glomerular filtration rate; N/A, not applicable.

**Table 4:** Multivariable Cox regression for risk factors for incident fractures of the kidney transplant recipients with diabetes mellitus as a continuous time-dependent coefficient.

	HR (95% CI)	*p*
Variables at baseline		
Recipient age	1.035 (1.007–1.064)	0.013
Pretransplant diabetes mellitus	1.115 (0.439–2.832)	0.818
Diabetes Mellitus × time	1.049 (1.007–1.094)	0.022
History of CVD	0.898 (0.442–1.824)	0.766
RRT before KT		
Hemodialysis	1.000 (Reference)	
Preemptive KT	0.358 (0.111–1.160)	0.087
Retransplant	2.100 (0.980–4.502)	0.057
IL-2 receptor antibody	1.134 (0.156–8.253)	0.901
Anti-thymocyte globulin	0.158 (0.021–1.181)	0.072
Variables at 6 months post-transplantation		
Hemoglobin	0.881 (0.762–1.018)	0.086
Phosphorus	0.722 (0.216–2.412)	0.596
Ca × P product	0.969 (0.762–1.018)	0.661
Vitamin D analogs	1.310 (0.701–2.448)	0.397
Tacrolimus dose	0.777 (0.546–1.104)	0.159
Tacrolimus dose per body weight	2.653 (0.373–18.883)	0.330
Tacrolimus trough level	1.112 (1.029–1.202)	0.008
Mycophenolic acid	2.224 (0.885–5.592)	0.089

CVD, cardiovascular disease; RRT, renal replacement therapy; KT, kidney transplantation; Ca, calcium; P, phosphorus; IL-2, interleukin-2.

## Data Availability

The data used in this study are available from the KOTRY office upon reasonable request.
